# Dangerous Liaison: Helicobacter pylori, Ganglionitis, and Myenteric Gastric Neurons: A Histopathological Study

**DOI:** 10.1155/2019/3085181

**Published:** 2019-12-30

**Authors:** Liana Sticlaru, Florica Stăniceanu, Mirela Cioplea, Luciana Nichita, Alexandra Bastian, Gianina Micu, Cristiana Popp

**Affiliations:** ^1^Pathology Department, Colentina University Hospital, Bucharest, Romania; ^2^“Carol Davila” University of Medicine and Pharmacy, Bucharest, Romania

## Abstract

Chronic inflammation induced by *Helicobacter pylori* (*H. pylori*) infection plays a major role in development of gastric cancer. However, recent findings suggested that progression of inflammation and neoplastic transformation in *H. pylori* infection are more complex than previously believed and could involve different factors that modulate gastric microenvironment and influence host-pathogen interaction. Among these factors, gastric myenteric plexus and its potential adaptive changes in H. pylori infection received little attention. This study is aimed at identifying the impact of *H. pylori*-associated gastritis on number and morphology of nerve cells in the stomach. The distribution of density, inflammation, and programmed cell death in neurons was immunohistochemically assessed in full-thickness archival tissue samples obtained from 40 patients with *H. pylori* infection who underwent surgery for gastric cancer and were compared with findings on samples collected from 40 age- and sex-matched subjects without bacteria. Overall, significant differences were noted between *H. pylori*-positive and *H. pylori*-negative patients. The analysis of tissue specimens obtained from those with infection revealed higher density and larger surface of the myenteric nervous plexus, as well as a significant increase in the number of gastric neuronal cell bodies and glial cells compared to controls. A predominant CD3-immunoreactive T cell infiltrate confined to the myenteric plexus was observed in infected subjects. The presence of mature B lymphocytes, plasma cells, and eosinophils was also noted, but to a lesser extent, within the ganglia. Myenteric ganglionitis was associated with degeneration and neuronal loss. Our results represent the first histopathological evidence supporting the hypothesis that *H. pylori*-induced gastric inflammation may induce morphological changes in myenteric gastric ganglia. These findings could help gain understanding of some still unclear aspects of pathogenesis of *H. pylori* infection, with the possibility of having broader implications for gastric cancer progression.

## 1. Introduction

*H. pylori* is one of the most widespread human pathogens and is the strongest known risk factor for malignancies arising within the stomach, mainly due to the persistent inflammatory response induced in the mucosa [1]. However, only a small proportion of colonized individuals develop gastritis and only a small subset of patients with chronic gastritis develop gastric cancer [2]. Furthermore, many of those with gastric inflammation are asymptomatic, while in some patients with overt gastritis, the symptoms persist or recur after eradication treatment [3]. This variability in clinical evolution could be explained by a number of host factors and bacterial virulence factors, but some authors suggested that the pathogenic mechanisms of *H. pylori* infection might be much more complex than generally believed and could involve some less studied individual factors, such as alterations of the gastric enteric nervous system (ENS) [4].

The ENS is by far the largest and most complex part of the autonomic nervous system (ANS), consisting of glial cells and various types of neurons organized in two networks of myenteric ganglia within the gut wall. It was described as the “brain in the gut,” since it has the unique ability to control gastrointestinal functions independent of the central nervous system [5]. In the stomach, the ENS is represented mainly by the Auerbach plexus (or the myenteric plexus), which is situated between the circular and the longitudinal layers of the muscularis propria and provides motor innervation to both muscle layers and secretomotor innervation to the gastric mucosa [6]. Only sparse submucosal ganglia, present mainly in the antrum, form the gastric Meissner plexus [7].

Some studies reported that gastric mucosal colonization by *H. pylori* and subsequent mucosal inflammation may affect central and extragastric peripheral nervous system activity, contributing to intestinal dysfunctions, cardiac arrhythmia, alterations of pancreatic function, and even to some neurological disorders, such as Parkinson's disease and Guillain-Barré syndrome [8–11]. On the other hand, other studies demonstrated that *H. pylori* infection plays a protective role against some esophageal diseases, inflammatory bowel diseases, Alzheimer's disease, and multiple sclerosis, resulting from changes induced in brain-gut axis [4, 11–15]. Based upon these observations, it is likely that *H. pylori* infection may also interact with gastric ENS through different mechanisms: direct neurotoxic effect and microelement deficiency, secondary to functional and morphological changes in the gastric mucosa, activation of neurogenic inflammation, and structural alterations of myenteric ganglia [4]. The last one is best suited for histomorphological and immunohistochemical approach.

Aside from *H. pylori*-associated changes in the neurochemical (neurotransmitter/neuropeptide) content of gastric nerve fibers, too few studies have been done to determine whether the H. pylori-induced gastric inflammation could cause neuroplastic alterations in the myenteric ganglia. Therefore, in this study, we aimed to directly assess the effects of *H. pylori* infection on gastric nervous system morphology, in order to shed light on the potential abnormalities that may result from it. Our hypothesis is that *H. pylori* infection influences the number of myenteric neurons and glial cells and disturbs neuronal homeostasis.

## 2. Materials and Methods

### 2.1. Patients and Tissue Samples

This study as designed as an observational retrospective cohort study following the methods previously published by our group [16]. Full-thickness samples of gastric wall were obtained from 40 consecutive patients (31 males, 9 females), mean age 63.43 (SEM = 1.86) undergoing surgery for gastric cancer. *H. pylori* infection was histologically proven in all the enrolled subjects. Archival gastric samples from 40 age- and sex-matched subjects (mean age 63.6, SEM = 1.75) without recent history of *H. pylori* infection, who had been operated for complicated peptic ulcer disease or non-adenocarcinomatous gastric tumors, served as controls and have been selected from same anatomical gastric region as ones of the *H. pylori*-positive group. All samples were harvested from areas at least 5 cm away from any visible lesion. Patients with peritonitis or suffering from different conditions associated with changes in myenteric plexus, as well as patients treated with chemo/radiotherapy, were excluded from the study. Moreover, subjects with morphologic evidence of recently treated *H. pylori* infection (prominent intestinal metaplasia, marked glandular atrophy, or nodular lymphoid aggregates in lamina propria) were not included in the control group.

### 2.2. Sample Processing and Histological Assessment

Routinely fixed and processed samples were cut in 5 *μ*m thick serial sections with circular layer and myenteric ganglia cut longitudinally. Three gastric cross sections per specimen, cut at a reasonable distance of 200 *μ*m, were mounted on glass slides and then examined. We took this measure to avoid evaluating the same ganglionic area twice in adjacent sections. Before use, slides were deparaffinized, rehydrated, and processed for routine hematoxylin and eosin (H&E) and Giemsa staining and immunohistochemistry. Histopathological findings were assessed on H&E-stained sections, and Giemsa stain technique was used to demonstrate *H. pylori*. The grades of *H. pylori* density, chronic mucosal inflammation, neutrophilic activity, intestinal metaplasia, and glandular atrophy were determined for each specimen and scored as normal, mild, moderate, and marked according to the updated Sydney system [17]. Neuron damage was confirmed when cells with condensed/vacuolated cytoplasm and/or shrunken, pyknotic nuclei were identified and was described as present/absent.

### 2.3. Immunohistochemical Analysis

Myenteric neurons and glial cells were evaluated by anti-HuC/D and anti-S100 antibodies, respectively. Ganglionic areas were measured by using anti-S100 antibody. Presence and quantification of lymphocytic infiltrate were assessed by using CD3 (T lymphocytes) and CD20 (B lymphocytes) antibodies. Apoptotic activity of myenteric neurons was examined with immunostaining using monoclonal human bcl-2 antibody. Antigen retrieval was performed in citrate buffer (pH 6.0) for HuC/D, whereas Tris-EDTA buffer was used for the rest of antibodies. All slides were microwaved at 500 W for 10 minutes. They were exposed to 3% hydrogen peroxide solution in order to block endogenous peroxidase activity. Sections were incubated with the respective antibodies at 4°C overnight (HuC/D) and for 30-60 minutes at room temperature (other antibodies). The bound antibody was visualized using biotinylated anti-rabbit or anti-mouse secondary antibody, and then streptavidin-peroxidase complex. Diaminobenzidine tetrahydrochloride was used as chromogen substrate. Slides were subsequently counterstained with Mayer's hematoxylin. For each antibody, all slides were simultaneously immunostained in order to rule out differences caused by the staining procedure.

### 2.4. Quantitative Assessment of Mucosal Inflammation

CD3 and CD20 lymphocytic mucosal inflammation was semiquantitatively graded on a 3-tier scale, according to the percentage of the area in the lamina propria infiltrated by inflammatory cells, as follows: grade 1 (5-30%), grade 2 (30-60%), and grade 3 (>60%). Lymphoid follicles were excluded from analysis, since their random distribution in the mucosa might otherwise generate less consistent results.

### 2.5. Quantitative Assessment of Myenteric Inflammation and Ganglion Cells

Evaluation of myenteric plexus inflammation was performed as described previously, according to the criteria proposed by Villanacci et al. [16, 18]. Briefly, we counted only T CD3+ cells the most severely inflamed ganglionic area and grade their density as mild (score 1—four or less lymphocytes observed), moderate (score 2—five to nine cells present), and marked (score 3—ten or more periganglionic lymphocytes identified).

In order to evaluate the immunoreactive ganglionic cells, we used a slightly modified version of a previously described method [19]. For each section, 40 sequential microscopic fields taken along the myenteric plexus were examined at 40x magnification, starting with the first ganglion present on the left side of the section. Examination of the sections and image acquisition were performed using an Olympus BX43 microscope equipped with an Olympus XC30 digital camera (Olympus Corporation, Japan) and ganglionic areas were estimated by an Image Analysis Software (cellSens Dimension, Olympus Corporation, Japan). Each microscopic field corresponded to a 0.36 mm × 0.27 mm rectangle, with an covered area of 0.0972 mm^2^. Thereby, the total ganglionic length and tissue area evaluated for each section were 14.4 mm and 3.888 mm^2^, respectively.

### 2.6. Statistical Analysis

For each patient, the results were expressed as mean ± SE. For groups, most data did not follow a parametric distribution, so they are presented using medians and interquartile ranges. The figures are designed as box-whiskers plots. The Wilcoxon test for nonparametric data (two-tailed) was performed to compare groups. The strength of association between variables was evaluated using the *γ*^2^ and Spearman rank correlation tests. A *p* value < 0.05 was considered statistically significant.

## 3. Results

On histological examination, there were 20 intestinal, 10 poor cohesive, 7 mixed, and 3 mucinous carcinoma subtypes, according to the 2019 WHO classification of gastric tumors [20]. Most tumors were located in the antrum, along the lesser curvature (27 cases), followed by body (11 cases) and cardia (2 cases). 23 cases were diagnosed as moderately differentiated carcinomas, with the remaining being poorly differentiated.

### 3.1. Gastric Mucosa

Most cases (22) showed a moderate degree of H. pylori colonization, while 13 cases had a mild bacterial density. In 5 cases, the presence of H. pylori was significant and scored as marked. All patients had chronic gastritis, and neutrophilic activity was observed in 31 (77.5%) of them. Immunohistochemical analysis revealed that the gastric mucosal inflammatory response consisted mainly of CD3+ T cells. Intestinal metaplasia and atrophy were observed in 25 and 21 patients, respectively.

### 3.2. Gastric Myenteric Plexus

**Figure 1:**
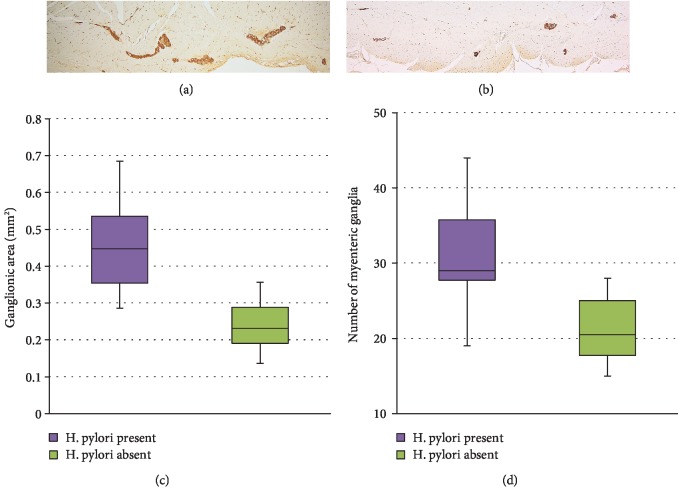
Number and area of myenteric ganglia in the stomach. Representative photomicrographs of S-100 immunostained ganglionic areas in H. pylori-positive patients (a, ×40) and in control patients (b, ×40). Box and whisker plots showing that gastric myenteric ganglia are larger (c, *p* < 0.01) and they are increased in number (d, *p* < 0.01) in H. pylori-infected patients, as compared to controls.

Ganglionic areas were significantly larger (median 0.447 mm^2^), and the number of myenteric ganglia was higher (median 29) in *H. pylori*-positive patients, compared to controls (medians 0.231 mm^2^ and 20.5, respectively, Figure 1).

**Figure 2:**
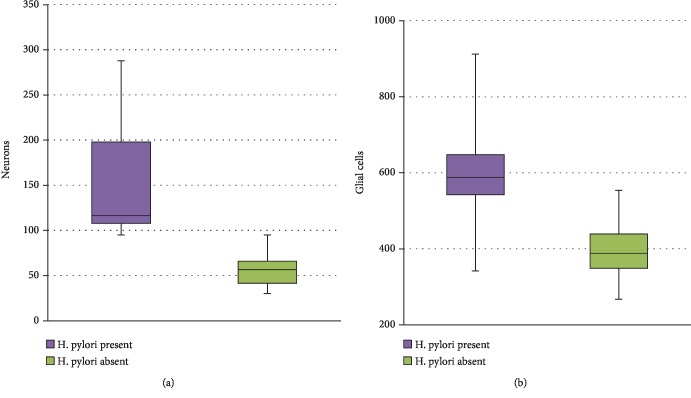
Number of gastric myenteric neurons and glial cells. Graphs showing that significant more myenteric neurons (a, *p* < 0.00001) and glial cells (b, *p* < 0.00001) were detected in the H. pylori-positive group in comparison to the control group.

An important difference was also found concerning the number of myenteric neurons between patients with *H. pylori*-induced gastritis (median 116.5) and those without infection (median 56.5) (Figure 2(a)), with a significant increment of +171% (individual values varying between 35% and 380%). In addition, more glial cells were identified in myenteric ganglia of infected patients (median 588) compared to controls (median 314) (Figure 2(b)), with a mean increment of +87% (individual values varying between 19% and 172%). Interestingly, in the control group, the number of ganglionic areas (median 20.5) and neuronal density (2-3 neurons per ganglionic area) did not correlate significantly with patients' age or with gastric region. The ratio between glial cells and neurons in myenteric plexus was fairly constant in *H. pylori*-negative patients, (range 5.1-6.8), whereas infected subjects did not display a correlation between glial and neuronal compartments, and the ratio was slightly decreased (range 2.6–6.3, *p* = 0.0151).

**Figure 3:**
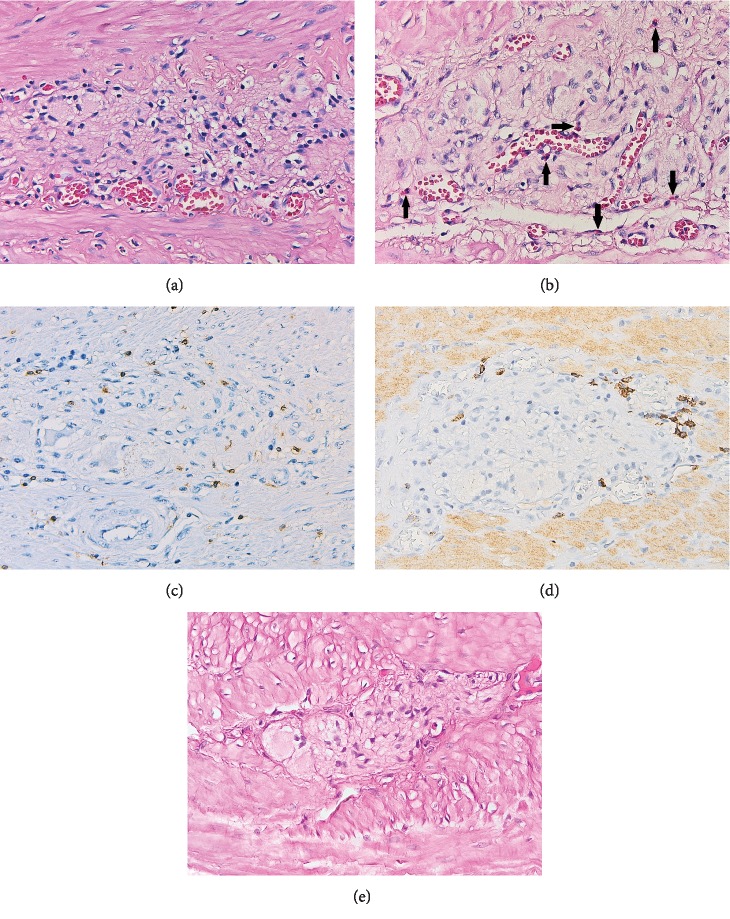
Representative photomicrographs showing different types of inflammatory cells around and within the myenteric plexus in H. pylori-infected patients: lymphocytes (a, H&E stain, 400x); lymphocytes and eosinophils (arrows) (b, H&E stain, 400x); T lymphocytes (c, CD3 stain, 400x); B lymphocytes (d, CD20 stain, 400x). In contrast, no inflammatory cell was noted around myenteric ganglia in control patients (e, H&E stain, 400x).

Ganglionitis was found in 33 (82.5%) cases with *H. pylori* infection. The inflammatory infiltrate was composed predominantly of CD3-positive T cells, with a minor prevalence of B lymphocytes, plasma cells, and rare eosinophils (Figure 3). T lymphocytic infiltration of myenteric plexus was mild in 17 patients, moderate in 10, and severe in 3 of them, and correlated with T cell density in lamina propria (*p* < 0.001). Occasional inflammatory cells, most of them eosinophils, were present in the vicinity of ganglionic areas in 20 (50%) uninfected patients. Neither CD20-positive B lymphocytes nor plasma cells were observed in the control group.

**Figure 4:**
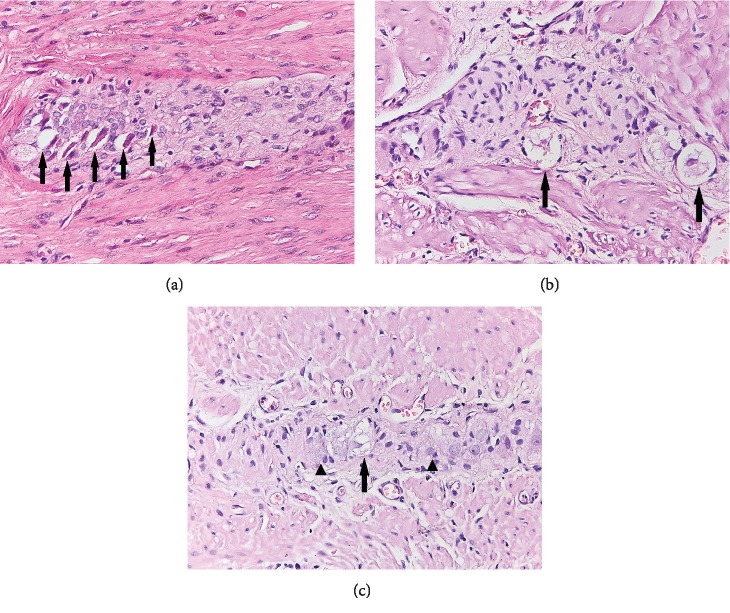
Representative photomicrographs illustrating signs of myenteric neuronal degeneration in H. pylori-positive patients: condensed cytoplasm and pyknotic nuclei (a, H&E stain, 400x) and vacuolated cytoplasm (b and c, H&E stain, 400x). Normal neurons are shown by arrowheads (c).

Degeneration of neuronal cells was obviously more frequently observed in *H. pylori*-infected patients (*p* < 0.0001, Figure 4) but was modestly correlated with T cell ganglionitis (*p* = 0.0306). However, a stronger association (*p* = 0.0024) was found between neurodegenerative changes and the polymorphous inflammatory infiltrate, including T and B lymphocytes and plasma cells.

**Figure 5:**
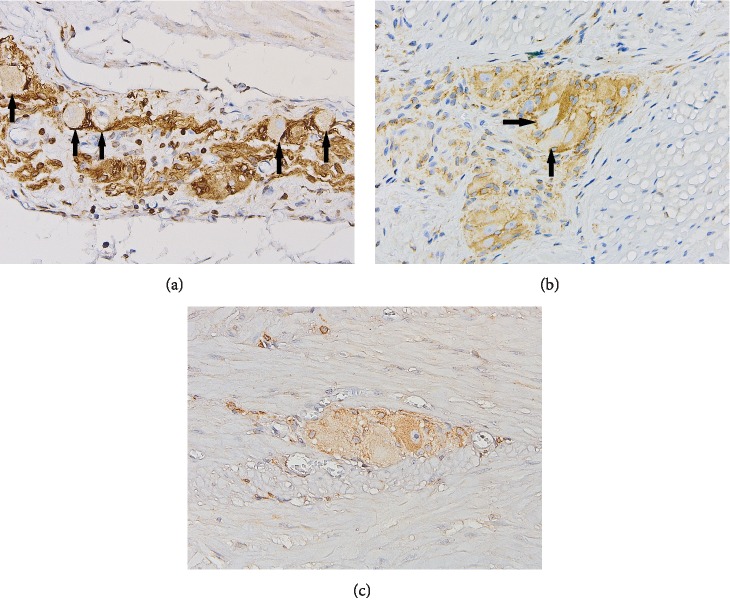
Bcl-2 immunohistochemical labeling of gastric myenteric ganglionic neurons: myenteric neurons with reduced or absent expression of antiapoptotic protein bcl-2 in H. pylori-positive patients (a and b, 400x) and normal bcl-2 expression of neurons in myenteric plexus from a control subject (c, 400x).

Myenteric neurons with markedly reduced or lost bcl-2 expression were observed in 23 (57.5%) infected patients, compared to only 3 (5.7%) cases in the control group (*p* < 0.0001, Figure 5). Neuronal apoptosis correlated with the presence of myenteric CD3-positive T cell infiltrate (*p* = 0.0056), but did not correlate with signs of neurodegeneration (*p* = 0.627).

## 4. Discussion

In the present study, we showed for the first time that the inflammatory process elicited by H. pylori colonization of gastric mucosa can cause inflammation of myenteric plexus and subsequently that myenteric ganglionitis induces structural changes in gastric myenteric ganglia. There is growing evidence that human enteric nervous system can be targeted by the immune response of the host in several chronic inflammatory digestive disorders [21–23]. Moreover, previous reports suggested that impaired neural activity might have a potential role in stomach cancer development [24–26].

### 4.1. Inflammation of Myenteric Plexus

The presence of periganglionic inflammation, referred to as enteric ganglionitis, or plexitis, reflects imbalanced neuroimmune interactions occurring within the enteric neural microenvironment [27]. In our study, the number of periganglionic inflammatory cells was significantly increased in *H. pylori*-positive patients compared to controls. Although this is an unusual finding, as gastritis is basically a mucosal disease, myenteric plexitis might be hypothesized as responsible for gastric dysmotility frequently described in H. pylori-induced gastritis [4, 28]. The immunohistochemical analysis of the myenteric infiltrate revealed a significant component of CD3-immunoreactive T cells, in agreement with previous reports showing that in inflammatory neuropathies there is a predominant T cytotoxic activity directed against proteins expressed by enteric neurons [18, 22]. However, in the present study, CD20-positive lymphocytes and plasma cells were exclusively identified in patients with *H. pylori* infection, indicating that, in addition to T lymphocyte activation, humoral immune response also participates in myenteric inflammation. Our results confirm previous data documenting the contribution of mature B cells to the immune response by synthesizing and releasing immunoglobulins directed against antigens expressed by myenteric neurons [27].

### 4.2. Myenteric Neuronal Degeneration and Apoptosis

Neuronal and nerve process degeneration in myenteric plexus has been documented in patients suffering from inflammatory bowel diseases. In our study, signs of neurodegeneration, such as vacuolated or condensed cytoplasm and pyknotic nuclei, were more frequently observed in infected patients, suggesting that *H. pylori* can induce neuronal damage in the myenteric plexus tissue. In addition, we observed a significant relationship between injury of myenteric neurons and periganglionic lymphoplasmacytic inflammatory infiltrate (*p* = 0.0024). However, a weaker correlation (*p* = 0.0306) with T cell myenteric infiltrate was also noted, indicating that degenerative changes of gastric neurons occur as a result of a concerted action of all the inflammatory cell types (including T cells, B cells, and plasma cells) recruited within myenteric plexus. Our observations confirm previous data showing the degeneration of myenteric neurons under enteric ganglionitis throughout the alimentary tract [29].

Bcl-2 antiapoptotic protein plays an essential role in protecting neurons from programmed cell death, promoting their survival in different types of neural injury. Our results showed, for the first time, that *H. pylori* is able to induce programmed cell death in myenteric gastric neurons. This finding is consistent with previous studies showing that *H. pylori* is able to promote apoptosis in infected gastric epithelial cells [30, 31] and leads to the conclusion that the bacteria might induce apoptosis dysregulation in different cell types of gastric wall. Moreover, we found a significant association between loss of bcl-2 expression in gastric neurons and periganglionic CD3-positive T lymphocytic infiltrate. This finding suggests that T cell-mediated immune response can trigger activation of the apoptotic pathways in myenteric neurons. This hypothesis is supported by similar observations in the central nervous system [32].

### 4.3. Neuronal and Glial Cell Hyperplasia

A very interesting and surprising finding in this study was the neuronal cell hyperplasia observed in patients with *H. pylori* infection. Variation in the number of enteric neurons was previously described by some authors in inflammatory bowel diseases [33, 34], while other studies failed to demonstrate any significant difference regarding neuron counting [19]. In the context of increased neuronal damage and apoptosis noted in infected patients, we are presently unable to explain the neuronal hyperplasia. In our opinion, the most reasonable hypothesis is that the increased number of gastric myenteric neurons represents a compensatory response to neuronal injury induced by ganglionic inflammation. However, although several possible pathways have been suggested [35–38], the mechanism underlying neuronal hyperplasia remains unknown. Further studies are necessary to elucidate if increased number of neuronal bodies is the result of proliferation and differentiation of neural crest-derived progenitors present in the gut or represents the consequence of transdifferentiation of mature enteric glial cells.

A significant increase in glial compartment was also detected by our analysis. Besides their traditional trophic and supportive functions for enteric neurons, glial cells are involved in enteric neurotransmission [21, 39], neurogenesis [40], and immune signaling [41, 42]; therefore, their number could be influenced by the immune response in the gastrointestinal tract. In our study, the level of neuronal hyperplasia was twice as great as glial cell hyperplasia degree, suggesting that neurons rather than glial cells were more affected in the *H. pylori*-positive patients herein examined. However, it is not clear if the proliferation of gastric glial cells precedes or follows neuronal hyperplasia.

Our study has some limitations. First, the number of patients was relatively small. Our results need to be verified in larger studies to obtain a more reliable estimation. Second, since this was a retrospective study, there may be a bias in the selection of patients, which we tried to minimize by examining 40 consecutive cases. Moreover, some risk factors that might have an impact on gastric myenteric plexus morphology, such as smoking and alcohol consumption, were not considered in this study, which may affect the reliability of the results. In addition, lack of prior research studies on the topic limits the robustness of our results. Future research should address these limitations to validate present findings.

In summary, the data presented provide what we believe is the first evidence that the gastric nervous system can be morphologically altered by host immune response in the setting of *H. pylori* infection. These findings advance our knowledge of the complex mechanisms of interaction between pathogen and host and will hopefully pave the road to a more vast scientific investigation in the area of gastric neural plasticity. Given the recognition of H. pylori as the major cause of gastric cancer, strategies aiming for a better understanding of the mechanisms of carcinogenesis are mandatory for identifying new potential therapeutic targets; therefore, further studies to clarify the involvement of the gastric enteric nervous system in gastric cancer development are needed.

## Data Availability

The data used to support the findings of this study are available from the corresponding authors upon reasonable request.

## References

[1] IARC (2012). *Monographs on the Evaluation of Carcinogenic Risks to Humans Volume 100b: A Review of Human Carcinogens: Biological Agents*.

[2] Bockerstett K. A., DiPaolo R. J. (2017). Regulation of gastric carcinogenesis by inflammatory cytokines. *Cellular and Molecular Gastroenterology and Hepatology*.

[3] Malfertheiner P., Megraud F., O'Morain C. A. (2012). Management of *Helicobacter pylori* infection–the Maastricht IV/Florence Consensus Report. *Gut*.

[4] Budzyński J., Kłopocka M. (2014). Brain-gut axis in the pathogenesis of *Helicobacter pylori* infection. *World Journal of Gastroenterology*.

[5] Goyal R. K., Hirano I. (1996). The enteric nervous system. *New England Journal of Medicine*.

[6] Furness J. B., Lloyd K. C., Sterini C., Walsh J. H. (1991). Evidence that myenteric neurons of the gastric corpus project to both the mucosa and the external muscle: myectomy operations on the canine stomach. *Cell and Tissue Research*.

[7] Furness J. B. (2006). *The enteric nervous system*.

[8] Domínguez-Muñoz J. E., Malfertheiner P. (2001). Effect of *Helicobacter pylori* infection on gastrointestinal motility, pancreatic secretion and hormone release in asymptomatic humans. *Scandinavian Journal of Gastroenterology*.

[9] Budzyński J., Kłopocka M., Bujak R., Swiatkowski M., Pulkowski G., Sinkiewicz W. (2004). Autonomic nervous function in *Helicobacter pylori*-infected patients with atypical chest pain studied by analysis of heart rate variability. *European Journal of Gastroenterology and Hepatology*.

[10] Nielsen H. H., Qiu J., Friis S., Wermuth L., Ritz B. (2012). Treatment for *Helicobacter pylori* infection and risk of Parkinson’s disease in Denmark. *European Journal of Neurology*.

[11] Kountouras J., Deretzi G., Zavos C. (2011). *Helicobacter pylori* infection may trigger Guillain -Barre syndrome, Fisher syndrome and Bickerstaff brainstem encephalitis. *Journal of the Neurological Sciences*.

[12] Rubenstein J. H., Inadomi J. M., Scheiman J. (2014). Association between *Helicobacter pylori* and Barrett’s esophagus, erosive esophagitis, and gastroesophageal reflux symptoms. *Clinical Gastroenterology and Hepatology*.

[13] Luther J., Dave M., Higgins P. D., Kao J. Y. (2010). Association between *Helicobacter pylori* infection and inflammatory bowel disease: a meta-analysis and systematic review of the literature. *Inflammatory Bowel Diseases*.

[14] Kountouras J., Boziki M., Gavalas E. (2009). Eradication of *Helicobacter pylori* may be beneficial in the management of Alzheimer’s disease. *Journal of Neurology*.

[15] Cook K. W., Crooks J., Hussain K. (2015). *Helicobacter pylori* infection reduces disease severity in an experimental model of multiple sclerosis. *Frontiers in Microbiology*.

[16] Sticlaru L., Stăniceanu F., Cioplea M. (2018). Neuroimmune cross-talk inHelicobacter pyloriinfection: analysis of substance P and vasoactive intestinal peptide expression in gastric enteric nervous system. *Journal of Immunoassay and Immunochemistry*.

[17] Dixon M. F., Genta R. M., Yardley J. H., Correa P. (1994). Classification and grading of gastritis: the updated Sydney system. International workshop on the histopathology of gastritis. *American Journal of Surgical Pathology*.

[18] Villanacci V., Bassotti G., Nascimbeni R. (2008). Enteric nervous system abnormalities in inflammatory bowel diseases. *Neurogastroenterology and Motility*.

[19] Ippolito C., Segnani C., de Giorgio R. (2009). Quantitative evaluation of myenteric ganglion cells in normal human left colon: implications for histopathological analysis. *Cell and Tissue Research*.

[20] Nagtegaal I. D., Odze R. D., Klimstra D. (2019). *WHO Classification of Tumors of the Digestive System*.

[21] Vasina V., Barbara G., Talamonti L. (2006). Enteric neuroplasticity evoked by inflammation. *Autonomic Neuroscience*.

[22] De Giorgio R., Guerrini S., Barbara G. (2004). Inflammatory neuropathies of the enteric nervous system. *Gastroenterology*.

[23] Barbara G., Cremon C., de Giorgio R. (2011). Mechanisms underlying visceral hypersensitivity in irritable bowel syndrome. *Current Gastroenterology Reports*.

[24] Polli-Lopes A. C., Zucoloto S., de Queirós Cunha F., da Silva Figueiredo L. A., Garcia S. B. (2003). Myenteric denervation reduces the incidence of gastric tumors in rats. *Cancer Letters*.

[25] Rosso M., Robles-Frías M. J., Coveñas R., Salinas-Martín M. V., Muñoz M. (2008). The NK-1 receptor is expressed in human primary gastric and colon adenocarcinomas and is involved in the antitumor action of L-733,060 and the mitogenic action of substance P on human gastrointestinal cancer cell lines. *Tumour Biology*.

[26] Feng F., Yang J., Tong L. (2011). Substance P immunoreactive nerve fibres are related to gastric cancer differentiation status and could promote proliferation and migration of gastric cancer cells. *Cell Biology International*.

[27] De Giorgio R., Camilleri M. (2004). Human enteric neuropathies: morphology and molecular pathology. *Neurogastroenterology and Motility*.

[28] Zhang C. L., Geng C. H., Yang Z. W. (2016). Changes in patients’ symptoms and gastric emptying after Helicobacter pylori treatment. *World Journal of Gastroenterology*.

[29] Demir I. E., Schäfer K.-H., Tieftrunk E., Friess H., Ceyhan G. O. (2013). Neural plasticity in the gastrointestinal tract: chronic inflammation, neurotrophic signals, and hypersensitivity. *Acta Neuropathologica*.

[30] El-Shahat M., El-Masry S., Lofty M., El-Kenawy A.-M., Nasif W. A. (2005). Relationship of Helicobacter pylori to Bcl-2 family expression, DNA content, and pathological characteristics of gastric cancer. *International Journal of Gastrointestinal Cancer*.

[31] Chu S. H., Lim J. W., Kim D. G., Lee E. S., Kim K. H., Kim H. (2011). Down-regulation of Bcl-2 is mediated by NF-*κ*B activation in helicobacter pylori-induced apoptosis of gastric epithelial cells. *Scandinavian Journal of Gastroenterology*.

[32] Aktas O., Ullrich O., Infante-Duarte C., Nitsch R., Zipp F. (2007). Neuronal damage in brain inflammation. *Archives of Neurology*.

[33] Bernardini N., Segnani C., Ippolito C. (2012). Immunohistochemical analysis of myenteric ganglia and interstitial cells of Cajal in ulcerative colitis. *Journal of Cellular and Molecular Medicine*.

[34] Geboes K., Collins S. (1998). Structural abnormalities of the nervous system in Crohn’s disease and ulcerative colitis. *Neurogastroenterology & Motility*.

[35] Metzger M., Bareiss P. M., Danker T. (2009). Expansion and differentiation of neural progenitors derived from the human adult enteric nervous system. *Gastroenterology*.

[36] Schuster A., Klotz M., Schwab T. (2014). Maintenance of the enteric stem cell niche by bacterial lipopolysaccharides? Evidence and perspectives. *Journal of Cellular and Molecular Medicine*.

[37] Belkind-Gerson J., Hotta R., Nagy N. (2015). Colitis induces enteric neurogenesis through a 5-HT4-dependent mechanism. *Inflammatory Bowel Diseases*.

[38] Belkind-Gerson J., Graham H. K., Reynolds J. (2017). Colitis promotes neuronal differentiation of Sox2+ and PLP1+ enteric cells. *Scientific reports*.

[39] Rühl A. (2005). Glial cells in the gut. *Neurogastroenterology and Motility*.

[40] Laranjeira C., Sandgren K., Kessaris N. (2011). Glial cells in the mouse enteric nervous system can undergo neurogenesis in response to injury. *The Journal of Clinical Investigation*.

[41] Geboes K., Rutgeerts P., Ectors N. (1992). Major histocompatibility class II expression on the small intestinal nervous system in Crohn's disease. *Gastroenterology*.

[42] Rühl A., Franzke S., Collins S. M., Stremmel W. (2001). Interleukin-6 expression and regulation in rat enteric glial cells. *American Journal of Physiology*.

[43] Cioplea M., Sticlaru L., Popp C., Micu G., Nichita L. (2018). Gastric myenteric plexus and Helicobacter pylori infection – is there a relationship?. *Virchows Archiv*.

